# [Retracted] Effects of miR-106b-3p on cell proliferation and epithelial-mesenchymal transition, and targeting of ZNRF3 in esophageal squamous cell carcinoma

**DOI:** 10.3892/ijmm.2025.5677

**Published:** 2025-10-27

**Authors:** Guanen Qiao, Chenguang Dai, Yang He, Junjie Shi, Chunfang Xu

Int J Mol Med 43: 1817-1829, 2019; DOI: 10.3892/ijmm.2019.4107

Following the publication of this paper, it was drawn to the Editor's attention by a concerned reader that certain of the western blot data shown in Fig. 8A and two panels of the Transwell assay data shown in Fig. 5B were strikingly similar to data that subsequently appeared in a pair of other publications. In addition, in Fig. 1C, the data panels shown for the KYSE150 and EC9706 cell lines, and also for the ECA-109 and HET-1A cell lines, were found to be overlapping, such that data which were intended to have shown the results from four cell lines appeared to have been derived from only two cell lines; in Fig. 2E, the same image was apparently included for the 'Control' colony formation assay experiments for the two different cell lines investigated (ECA-109 and KYSE150); and possible anomalies were identified with the western blot data in Fig. 3C.

After having performed an independent review of the data in the Editorial Office, the Editor of *International Journal of Molecular Medicine* has decided that this paper should be retracted from the Journal on account of a lack of confidence in the presented data. The authors were asked for an explanation to account for these concerns, but the Editorial Office did not receive a reply. The Editor apologizes to the readership for any inconvenience caused.

